# Effects of Post-Anthesis Nitrogen Uptake and Translocation on Photosynthetic Production and Rice Yield

**DOI:** 10.1038/s41598-018-31267-y

**Published:** 2018-08-27

**Authors:** Hui Wu, Jing Xiang, Yuping Zhang, Yikai Zhang, Shaobing Peng, Huizhe Chen, Defeng Zhu

**Affiliations:** 1 State Key Laboratory of Rice Biology, China National Rice Research Institute, Hangzhou, 311400 P.R. China; 2 National Key Laboratory of Crop Genetic Improvement, MOA Key Laboratory of Crop Ecophysiology and Farming System in the Middle Reaches of the Yangtze River, College of Plant Science and Technology, Huazhong Agricultural University, Wuhan, Hubei 430070 China

## Abstract

Post-anthesis nitrogen uptake and translocation play critical roles in photosynthetic assimilation and grain filling. However, their effects on leaf stay-green characteristics, dry matter accumulation, and translocation after anthesis remain unclear. In this study, post-anthesis N uptake and translocation between two different rice genotypes (Yongyou12 and Zhongzheyou1) were compared through soil nitrogen leaching treatments at the meiosis stage (MST) and anthesis stage(AST) respectively, and their effects on leaf stay-green duration, photosynthesis, dry matter accumulation and translocation during ripening and yield formation were estimated. The results showed that the soil nitrate-N and ammonium-N contents in Yongyou12 pots decreased significantly, and post-anthesis N uptake was 2.0–3.4 fold higher in Yongyou12 than in Zhongzheyou1. The activities of N-metabolism enzymes and antioxidant enzymes were higher, and flag-leaf photosynthesis and dry matter accumulation during ripening were greater, in Yongyou12 than in Zhongzheyou1. However, insufficient available soil N led to significant decreases in the activities of N- metabolism enzymes, decreased flag-leaf photosynthesis, increased translocation of dry matter and N pre-anthesis, accelerated leaf senescence, shorter duration of the leaf stay-green period, and decreased dry matter accumulation and grain plumpness. In addition, the effect of N uptake after anthesis on yield is greater for rice genotypes that depend on post-anthesis dry matter accumulation and an expanded sink capacity.

## Introduction

Rice (*Oryza sativa* L.) is one of the most important cereal crops globally, being the primary food source for more than half the world’s population^1,2^. Grain filling, a crucial determinant of grain yield and rice productivity, is characterized by the duration and rate of grain filling, and varies widely among genotypes. It has been reported that a longer period of grain filling, which leads to higher cumulative mean temperature and cumulative solar radiation, leads to greater dry matter accumulation and is the main determinant of grain yield^3,4^. Park and Lee proposed that leaf stay-green characteristics and delayed senescence of upper leaves would contribute to increasing grain yield through improved photosynthesis during the grain-filling period^5,6^. At present, super rice, especially inter-subspecific hybrid rice cultivars, have characteristics of large dry matter accumulation after anthesis, a long grain-filling period (>60 d), and large yield potential in China^7,8^.

Nitrogen (N) fertilizer is one of the most important agronomic inputs and a limiting factor for realizing potential grain production, and high-yield productivity of rice is usually accompanied by greater N accumulation^9,10^. Positive correlations have been found between grain yield and N accumulation after heading, suggesting that increasing N accumulation after heading is crucial to increase grain yields^11^. Meanwhile, the accumulation of N in upper leaves after anthesis is also vital for maintaining the stay-green state of rice leaves^12^. Ribulose-1,5-bisphosphate carboxylase/oxygenase (Rubisco) in leaves accumulates to a level in excess of photosynthetic requirements, and serves as a store of N for grain filling^13^. The insufficient accumulation of N in the leaves and high N translocation from leaves after anthesis can accelerate leaf senescence, weaken the leaf photosynthetic capacity, and ultimately result in less assimilates for grain filling^14^.

The uptake and assimilation of N is achieved via an N-metabolizing enzyme pathway in which nitrate reductase (NR), the glutamine synthetase/glutamate synthase (GS/GOGAT) cycle and glutamate dehydrogenase (GDH) play important roles^15^. The regulation of N uptake by N-metabolism enzymes can not only enhance the photosynthetic capacity of leaves, but also prolong the stay-green duration of leaves, so that N-metabolism enzymes remain present and active in the leaves for longer. This seems to be a beneficial cycle. In addition, N deficiency or excess increase the production of reactive oxygen species (ROS) in plants, which results in lipid peroxidation of cell membranes^16^, the senescence and even death of plants. The antioxidant enzyme system plays an important role in scavenging ROS, delaying leaf senescence and prolonging the stay-green period of leaves^17,18^.

Although the relationships among dry matter, N accumulation and translocation, and rice yield have been studied^4,8,19^, more attention was focus on the nitrogen transport and leaf senescence^6,20,21^, as well as models of nitrogen accumulation after anthesis^22^, little information is available on the effects of post-anthesis N uptake and translocation on leaf stay-green characteristics, photosynthetic production, and yield in different rice genotypes. *Indica-Japonica* hybrid rice Yongyou12 (YY12) and *Indica* hybrid rice Zhongzheyou1 (ZZY1), were selected in this study, which are super rice varieties with high yield and large panicles and widely planted in the middle and lower reaches of the Yangtze River in China^4,23^. The main objectives of this study were to investigate the difference in post-anthesis N uptake and translocation between two rice genotypes and their effects on leaf stay-green duration, dry matter assimilation and redistribution after anthesis, and grain yield formation. Finally, a schematic diagram incorporating the physiological roles of N metabolism and antioxidant enzymes is presented to explain the mechanisms by which post-anthesis N uptake and translocation and leaf stay-green duration affect grain yield development.

## Results

### Dynamics of nitrogen content in soil and plant

**Figure 1:**
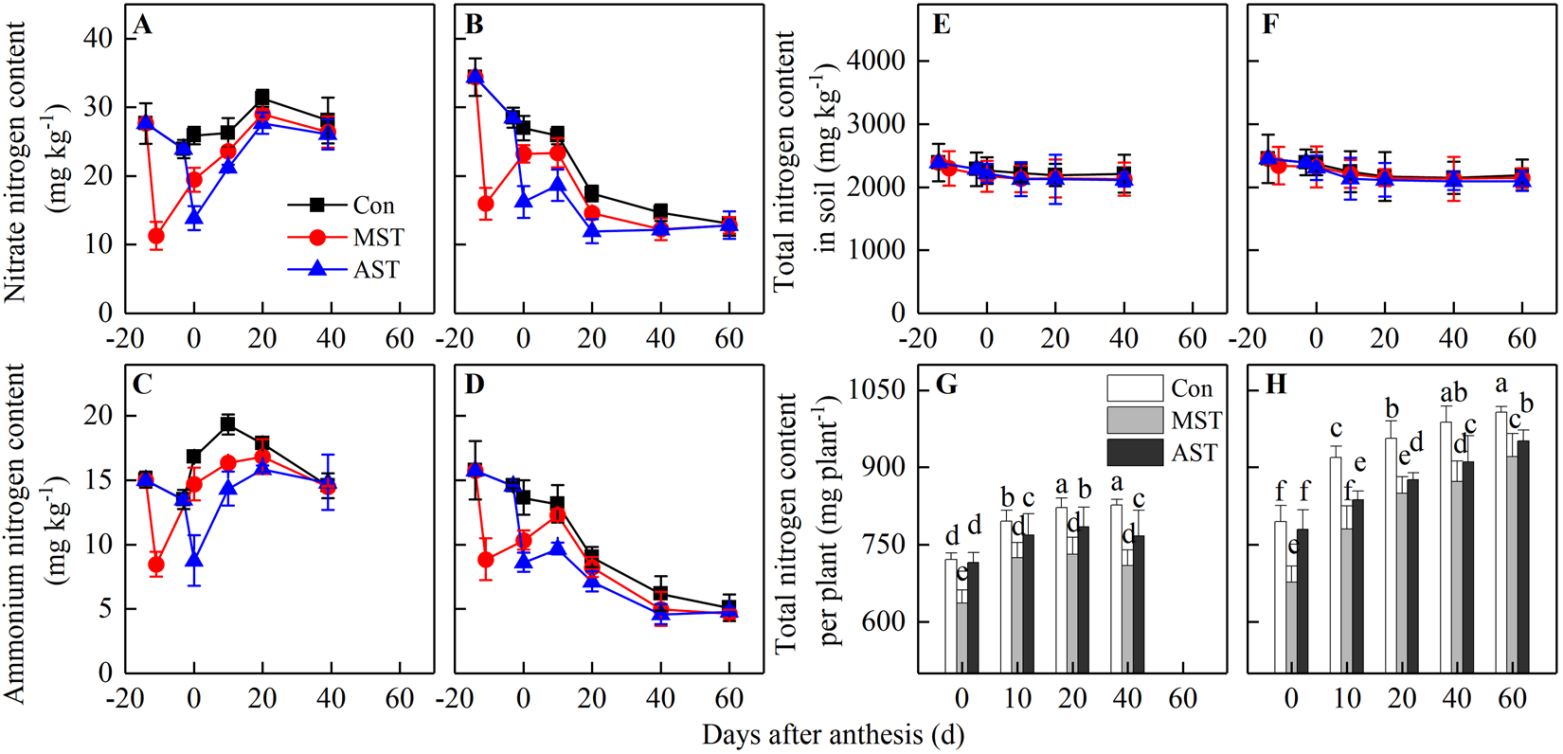
The changes of nitrate nitrogen, ammonium nitrogen and total nitrogen content in soil and total nitrogen content per plant after soil leaching in 2016. MST: leaching treatment at meiosis stage, 14^th^ day before anthesis; AST: leaching treatment at anthesis stage; Con: control, plants without leaching. Values are means ± SE of five pots (five replicates) of Zhongzheyou1 (**A**,**C**,**E**,**G**) and Yongyou12 (**B**,**D**,**F**,**H**). Different letters on columns indicate significant difference (*p* < 0.05, Tukey’s multiple test).

Two different soil N-leaching treatments were applied; one at the meiosis stage (MST, starting from the 14^th^ day before anthesis), and one at anthesis (AST, starting from the 3^rd^ day before anthesis). Plants in the control group were not subjected to a leaching treatment. Both of the soil N-leaching treatments significantly affected the amounts of nitrate-N and ammonium-N in soil around the two rice genotypes, but the total soil N content was unchanged (Fig. 1E,F). The contents of nitrate-N and ammonium-N in soil decreased rapidly and remarkably by 53.6–59.3% and 43.5–43.9% in the MST treatment, and by 42.1–43.1% and 35.0–40.9% in the AST treatment, compared with the control (no soil N-leaching). This effectively decreased the soil available N supply. After anthesis, the soil nitrate-N and ammonium-N contents in ZZY1 pots gradually increased, and the treatments could be ranked, from highest soil nitrate-N and ammonium-N contents to lowest, as follows: control > MST > AST (Fig. 1A,C). During grain filling, the soil available N content again decreased, and there were no significant differences among the treatments. In contrast, the soil available N content clearly decreased in YY12 pots from leaching to maturity, except for a slight increase within 10 days after anthesis (Fig. 1B,D). Similarly, the soil available N content was highest in the YY12 control pots, followed those in the MST treatment and finally those in the AST treatment. At the same time, the total nitrogen content of rice plant was decreased significantly under soil leaching treatments, and the treatments could be ranked, from highest plant nitrogen content to lowest, as follows: control > AST > MST (Fig. 1G,H). In addition, the total nitrogen accumulation per plant of YY12 was significantly higher than that of ZZY1.

### Activities of nitrogen-metabolism enzymes

**Figure 2:**
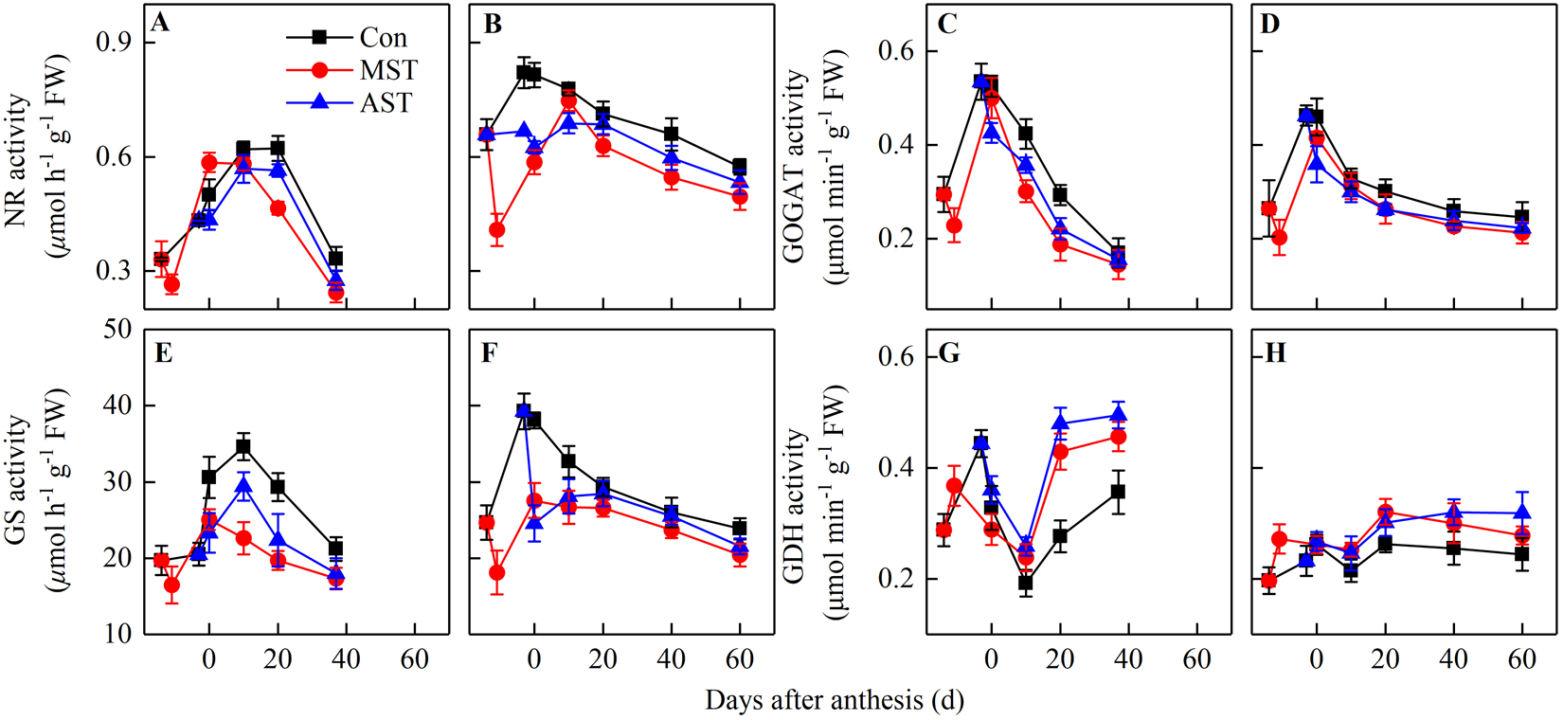
Nitrate reductase (NR), glutamine synthase (GS), glutamate synthase (NADH–GOGAT) and glutamate dehydrogenase (GDH) activities in the flag leaf from soil leaching to maturity. Values are means ± SE of five pots (five replicates) of Zhongzheyou1 (**A**,**C**,**E**,**G**) and Yongyou12 (**B**,**D**,**F**,**H**).

The activities of NR, GS, and NADH-GOGAT in the flag leaf exhibited the same trend in both rice genotypes. Their activities decreased markedly during soil leaching in the MST treatment, but were not significantly affected by soil leaching in the AST treatment (Fig. 2). From the meiosis stage to the maturity stage, the activities of NR, GS, and NADH-GOGAT first increased rapidly, and then gradually decreased during leaf senescence. In both genotypes, the treatments could be ranked, from highest enzyme activities to lowest, as follows: control > AST > MST. The activities of all three enzymes were higher in YY12 than in ZZY1 under the same treatment (Fig. 2A–F). In contrast, GDH activity was lowest from before anthesis to 10 days after anthesis, and then increased during leaf senescence. The treatments could be ranked, from highest GDH activity to lowest, as follows AST > MST > Control. The GDH activity was lower in YY12 than in ZZY1 regardless of the leaching period (Fig. 2G,H).

### Differences in photosynthetic capacity

**Figure 3:**
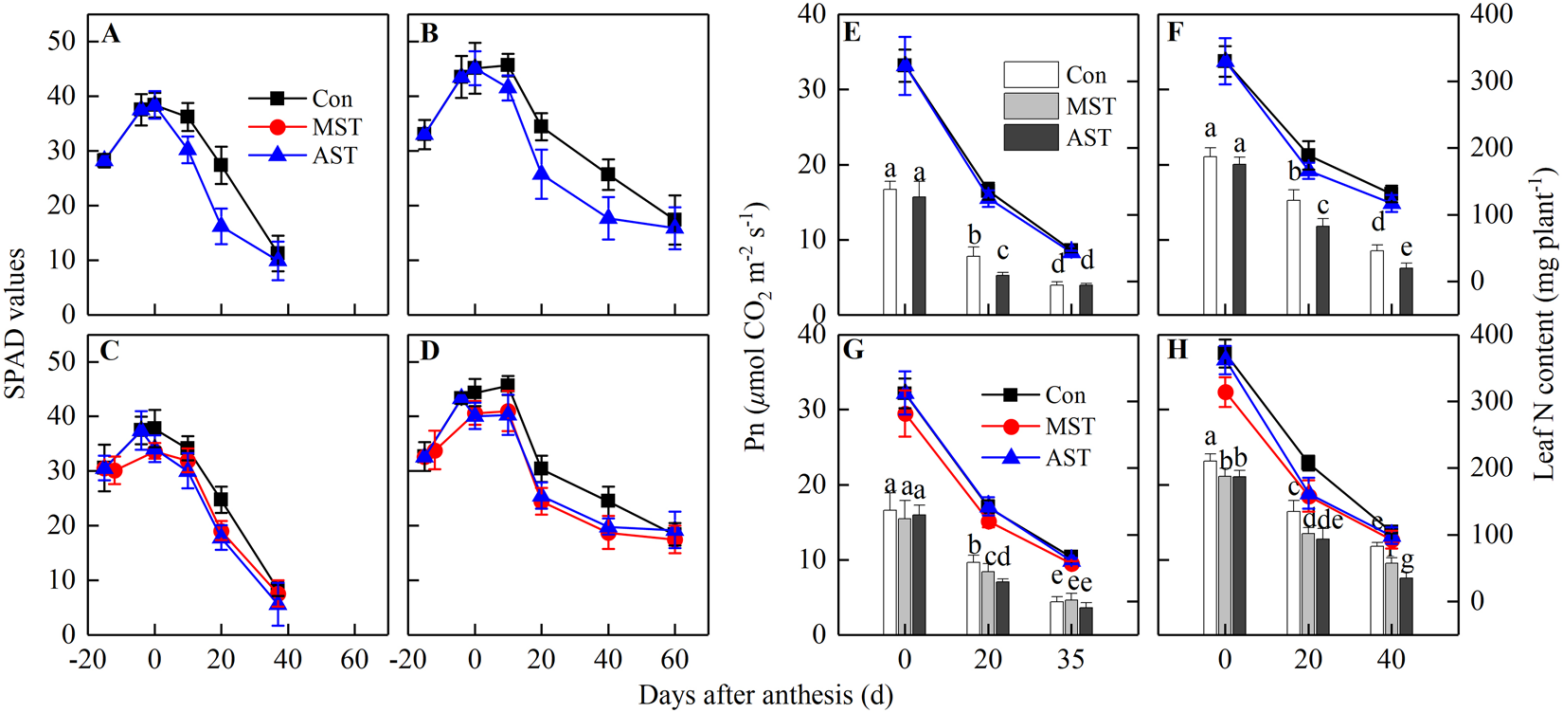
Effects of different leaching treatments on SPAD value and net photosynthesis rate (Pn) of flag leaf, and leaf N content in two years. SPAD values are means ± SE of 10 flag leaves of Zhongzheyou1 (**A**,**C**) and Yongyou12 (**B**,**D**) in 2015 (**A**,**B**) and 2016 (**C**,**D**), respectively. Pn values are means ± SE of 5 flag leaves of Zhongzheyou1 (**E**,**G**) and Yongyou12 (**F**,**H**) in 2015 (**E**,**F**) and 2016 (**G**,**H**), respectively. Leaf N content are means ± SE of five plants of Zhongzheyou1 (**E**,**G**) and Yongyou12 (**F**,**H**) in 2015 (**E**,**F**) and 2016 (**G**,**H**), respectively, which were sampled and measured synchronously with Pn. Within the same year, different letters on columns indicate significant difference (*p* < 0.05, Tukey’s multiple test).

Regardless of the leaching period or rice genotype, leaf SPAD decreased markedly during soil N leaching until grain maturity. The differences in leaf SPAD between MST and AST were not significant (Fig. 3A–D). A similar trend was observed for Pn and leaf N content, but the differences among the three treatments were more pronounced than those observed for SPAD values (Fig. 3E–H). On the whole, the AST treatment had the greatest impact on leaf Pn during the grain filling period, followed by the MST treatment. The SPAD, leaf N content and Pn values were higher in YY12 leaves than in ZZY1 leaves in each treatment.

### Activities of antioxidant enzymes

**Figure 4:**
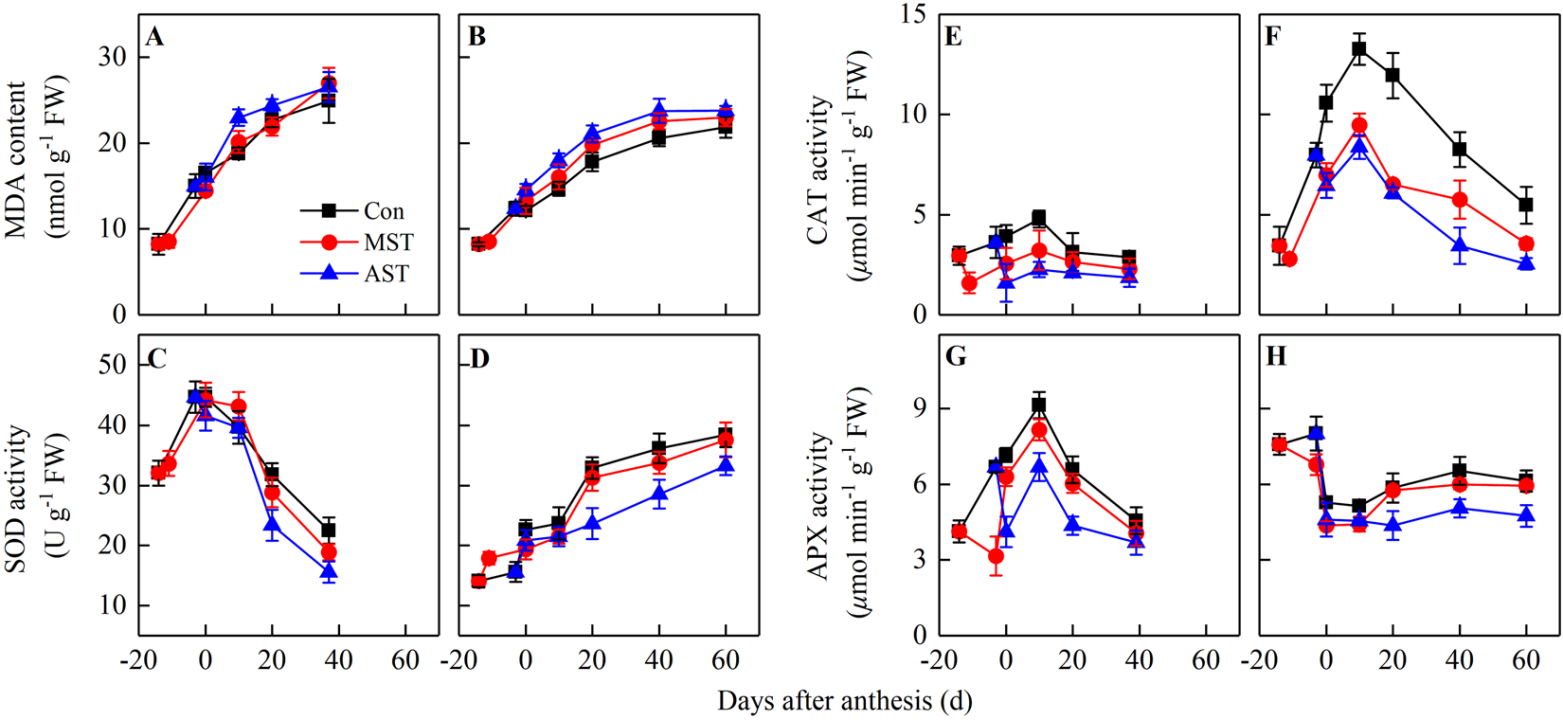
Changes in malondialdehyde (MDA) content, superoxide dismutase (SOD), catalase (CAT), and ascorbate peroxidase (APX) activities in the flag leaf from soil leaching to maturity. Values are means ± SE of five pots (five replicates) of Zhongzheyou1 (**A**,**C**,**E**,**G**) and Yongyou12 (**B**,**D**,**F**,**H**).

The malondialdehyde (MDA) content increased rapidly during leaf senescence. Soil N-leaching accelerated this trend, regardless of whether the leaching occurred pre- or post-anthesis (Fig. 4A,B). Soil N-leaching at different periods also affected the antioxidant enzyme activities in the two rice genotypes. The superoxide dismutase (SOD) activity in ZZY1 flag leaves increased rapidly before anthesis, even when soil N-leaching occurred at the meiosis stage, but decreased continuously during leaf senescence. These effects were stronger in the AST treatment than in the MST treatment (Fig. 4C). In contrast, the SOD activity in YY12 leaves markedly increased during ripening. The treatments could be ranked, from highest SOD activity to lowest, as follows: control > MST > AST (Fig. 4D). The CAT activity in the two genotypes also increased significantly after soil leaching, peaked at 10 days post-anthesis, and then decreased quickly. The CAT activity was highest in the control, followed by the MST treatment and then the AST treatment (Fig. 4E,F). Similar to SOD activity in ZZY1 leaves, APX activity in ZZY1 leaves first increased and then decreased (Fig. 4G). In contrast, APX activity in YY12 leaves first decreased and then increased (Fig. 4H). Overall, the inhibition of antioxidant enzyme activities and the increase in MDA content were more pronounced in the AST treatment than in the MST treatment, and YY12 always had lower MDA levels and higher antioxidant enzyme activities than did ZZY1.

### Characteristics of nitrogen uptake and translocation

**Figure 5:**
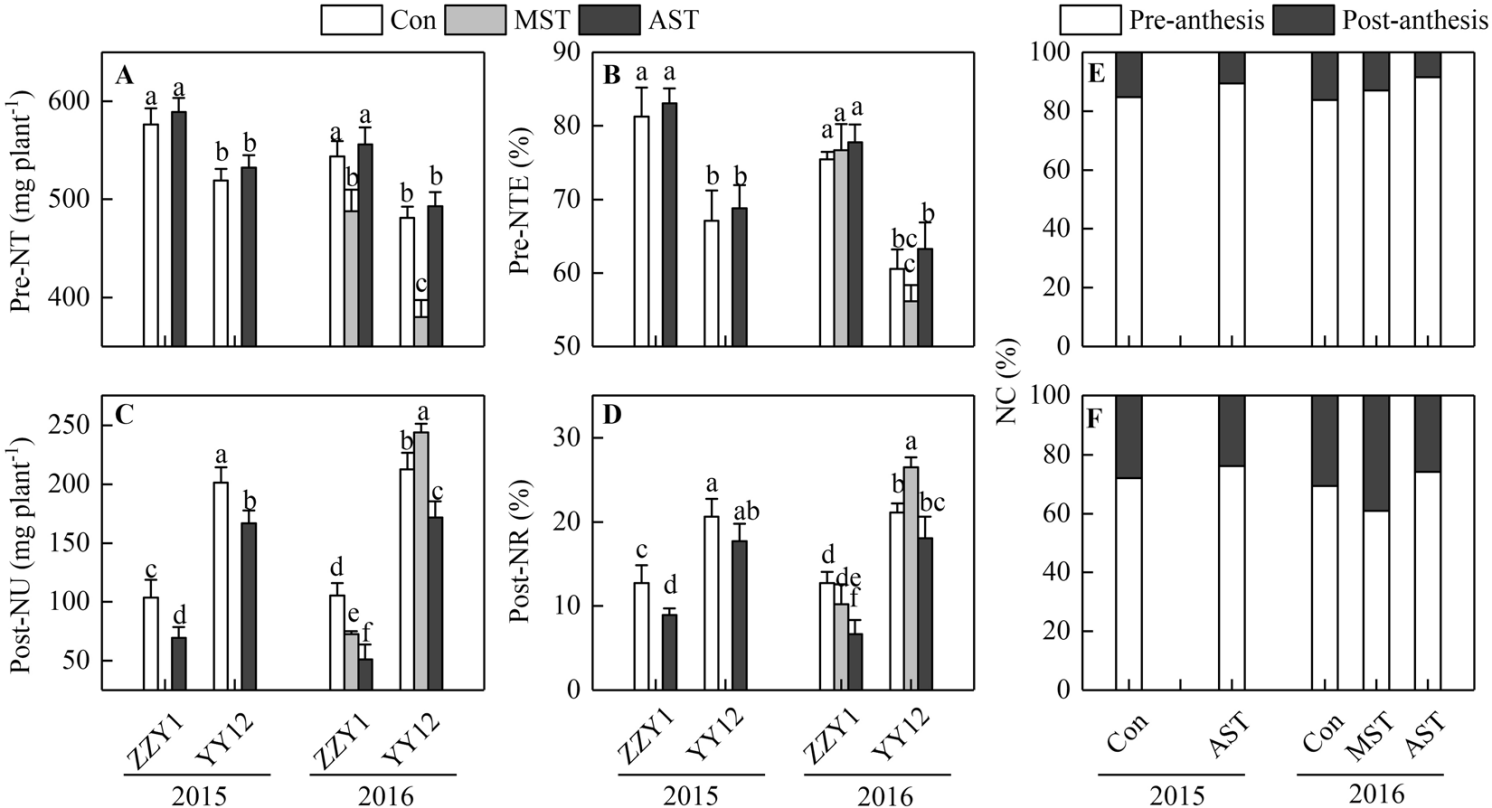
Characteristics of nitrogen uptake and translocation in rice subjected to leaching treatments in 2015 and 2016. (**A**) Pre-NT, pre-anthesis nitrogen translocation. (**B**) Pre-NTE, pre-anthesis nitrogen translocation efficiency. (**C**) Post-NU, post-anthesis nitrogen uptake. (**D**) Post-NR, ratio of Post-NU to total nitrogen accumulation. (**E**,**F**) NC, contribution of Pre-NT or Post-NU to grain nitrogen in Zhongzheyou1 (**E**) and Yongyou12 (**F**), respectively. Values are means ± SE of five pots (five replicates). Within the same year, different letters on columns indicate significant difference among leaching treatments and varieties (*p* < 0.05, Tukey’s multiple test).

There were significant differences in pre-anthesis translocation and post-anthesis N uptake between the two rice genotypes and between the two N-leaching treatments (Fig. 5). The pre-anthesis N translocation (Pre-NT) and pre-anthesis N translocation efficiency (Pre-NTE) were significantly lower in YY12 than in ZZY1 (Fig. 5A,B). In contrast, the post-anthesis N uptake (Post-NU) and the ratio of Post-NU to total N accumulation (Post-NR) were higher in YY12 than in ZZY1 (Fig. 5C,D). Compared with the control, the MST treatment resulted in significant decreases in Pre-NT, by 10.3% ZZY1 and by 21.0% in YY12. The Pre-NTE of YY12 also decreased in the MST treatment, but that of ZZY1 showed little change. The AST treatment slightly increased the Pre-NT and Pre-NTE of both rice varieties. The Post-NU decreased by 31.1% in ZZY1 in the MST treatment, and its Post-NR also decreased. In contrast, the Post-NU of YY12 increased by 14.7% in the MST treatment. Both Post-NU and Post-NR in the two genotypes decreased significantly in the AST treatment. Furthermore, the contribution of Pre-NT to grain N (pre-anthesis NC) in YY12 decreased sharply in the MST treatment, and the contribution of Post-NU to grain N (post-anthesis NC) increased significantly, whereas the opposite trend was observed for ZZY1 (Fig. 5E,F). In the AST treatment, the pre-anthesis NC increased in both genotypes, while the post-anthesis NC decreased. These effects were greater for YY12 than for ZZY1.

### Characteristics of dry matter accumulation and translocation

**Figure 6:**
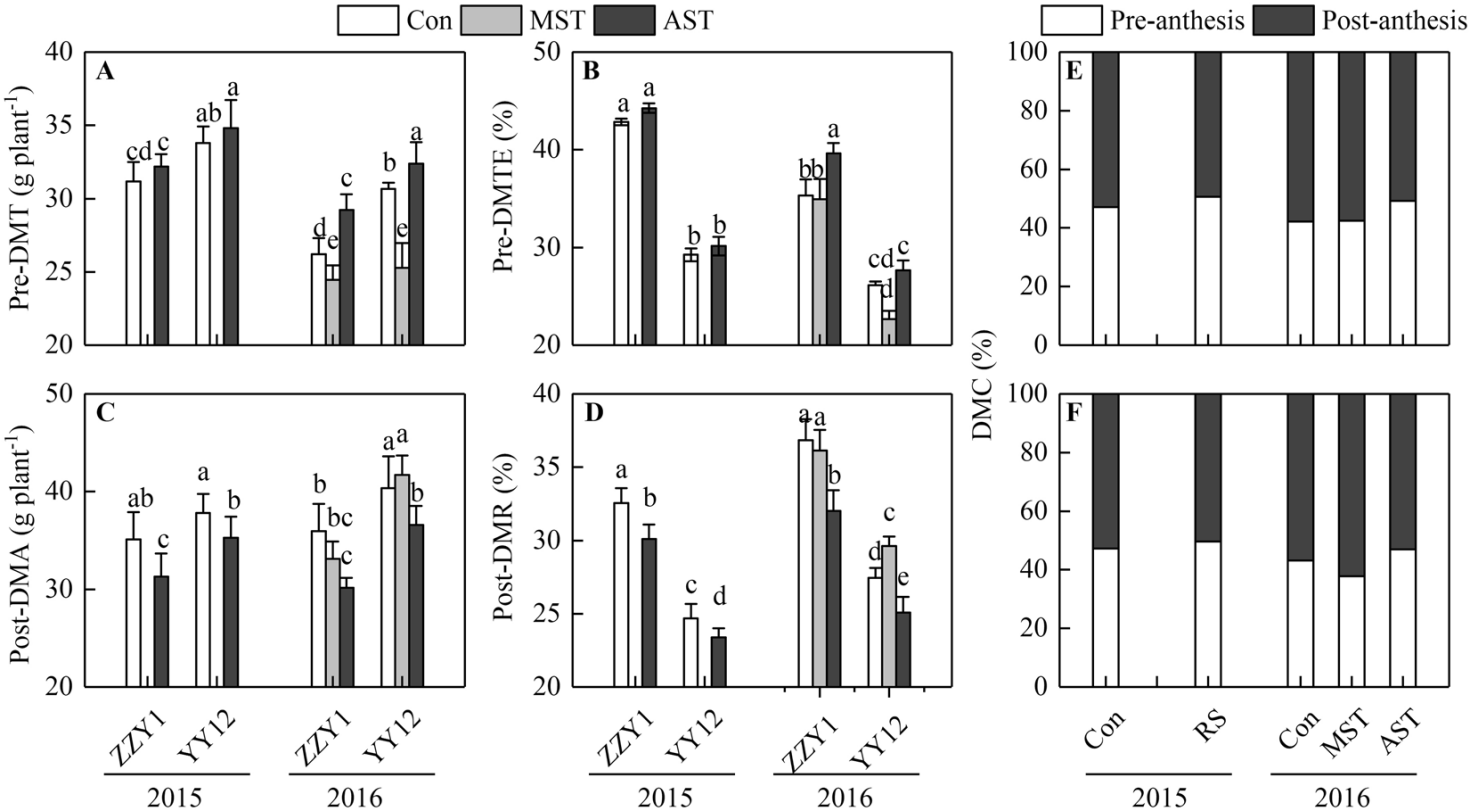
Characteristics of dry matter accumulation and translocation subjected to leaching treatments in 2015 and 2016. (**A**) Pre-DMT, pre-anthesis dry matter translocation. (**B**) Pre-DMTE, pre-anthesis dry matter translocation efficiency. (**C**) Post-DMA, post-anthesis dry matter accumulation. (**D**) Post-DMR, ratio of Post-DMA to total dry matter accumulation. (**E**,**F**) DMC, contribution of Pre-DMT or Post-DMA to grain yield in Zhongzheyou1 (**E**) and Yongyou12 (**F**), respectively. Values are means ± SE of five pots (five replicates). Within the same year, different letters on columns indicate significant difference among leaching treatments and varieties (*p* < 0.05, Tukey’s multiple test).

The pre-anthesis dry matter translocation (Pre-DMT) and post-anthesis dry matter accumulation (Post-DMA) were significantly higher in YY12 than in ZZY1 (Fig. 6A,C), but the pre-anthesis dry matter translocation efficiency (Pre-DMTE) and the ratio of Post-DMA to total dry matter accumulation (Post-DMR) were much lower in YY12 because of its large biomass (Fig. 6B,D). Similar to the characteristics of N uptake and translocation, the Pre-DMT and Pre-DMTE in both varieties and the Post-DMA and Post-DMR in ZZY1 declined under the MST treatment. The Post-DMA and Post-DMR in YY12 increased under the MST treatment, which resulted in a remarkable increase in post-anthesis DMC (contribution of Post-DMA to grain yield) in YY12 (Fig. 6F). At the same time, the Pre-DMT and Pre-DMTE of both genotypes slightly increased in the AST treatment, while the Post-DMA and Post-DMR decreased significantly. This led to an obvious decrease in post-anthesis DMC both genotypes. The AST treatment had a stronger effect on the post-anthesis DMC in ZZY1 than in YY12 (Fig. 6E,F).

### Leaf area, grain yield and its components

Since the leaching treatments were carried out only after plant leaves and plant types had been shaped, the effect of leaching on leaf area was negligible and there was no significant difference among different treatments. (Table 1). At the same time, the difference in total dry mass per plant did not reach a significant level among three treatments. The yield and its components were strongly affected by the N-leaching treatments and differed between the two rice genotypes in 2015 and 2016. In the MST treatment, the grain yield decreased significantly by 6.2% (ZZY1) and 10.4% (YY12). ZZY1 mainly showed a significant decrease in 1000-grain weight and seed-setting rate, while YY12 showed a marked decrease in 1000-grain weight and number of spikelets per panicle. There were significant differences in grain yield and 1000-grain weight of YY12 between the AST treatment and the control. However, there was no apparent difference in the yield of ZZY1 between the AST treatment and the control, despite a significant reduction in its 1000-grain weight. Overall, the grain yield of YY12 was significantly higher than that of ZZY1 (by 6.0–8.4%), while soil N-leaching had a greater impact on the yield of YY12.

**Table 1:** Effects of nitrogen leaching on total dry mass and leaf area at anthesis stage, grain yield and its components of two rice cultivars in two years.

Year	Varieties	Treatments	Total dry mass (g plant^−1^)	Leaf area (cm^2^ plant^−1^)	Spikelets per panicle	Seed-setting rate (%)	1000-grain weight (g)	Yield (g plant^−1^)
2015	Zhongzheyou1	Con	107.8 ± 6.3a	2988.7 ± 75.3a	218.8 ± 7.8a	93.1 ± 1.0a	27.0 ± 0.3a	63.3 ± 0.7a
		AST	104.1 ± 7.5a	2947.0 ± 51.2a	212.5 ± 13.5a	92.5 ± 0.7a	25.7 ± 0.4b	61.6 ± 1.3a
	Yongyou12	Con	153.3 ± 14.9a	2393.7 ± 45.1a	369.4 ± 9.3a	86.2 ± 0.7a	21.8 ± 0.3a	68.0 ± 0.6a
		AST	150.8 ± 16.6a	2499.0 ± 65.4a	368.7 ± 6.1a	87.3 ± 0.5a	20.4 ± 0.1b	64.4 ± 0.6b
2016	Zhongzheyou1	Con	110.2 ± 7.6a	2938.7 ± 43.9a	209.1 ± 3.7a	86.8 ± 1.1a	26.5 ± 0.3a	58.9 ± 0.8a
		MST	103.2 ± 8.0a	2907.0 ± 55.7a	210.4 ± 3.7a	81.3 ± 1.7b	25.7 ± 0.3b	55.3 ± 0.8b
		AST	103.9 ± 8.1a	2868.7 ± 34.1a	206.0 ± 6.6a	86.5 ± 1.1a	25.8 ± 0.1b	56.7 ± 0.7ab
	Yongyou12	Con	157.6 ± 14.0a	2573.7 ± 54.1a	386.6 ± 6.1a	82.9 ± 0.6a	21.9 ± 0.4a	65.3 ± 1.5a
		MST	153.2 ± 15.1a	2649.0 ± 66.5a	363.3 ± 5.2b	81.0 ± 1.5a	20.7 ± 0.1b	58.5 ± 0.9c
		AST	153.8 ± 18.0a	2599.7 ± 65.7a	391.1 ± 4.3a	82.2 ± 1.1a	20.7 ± 0.1b	61.6 ± 0.7b

Values are means ± SE of five pots (five replicates). Values followed by different letters are significantly different among leaching treatments (*p* < 0.05, Tukey’s multiple test).

## Discussion

Grain yield is determined by the translocation of dry matter stored in vegetative organs before anthesis and the accumulation of photosynthates after anthesis^24^. More than 60% of the assimilates for grain filling are generated through current photosynthesis^25–27^. In this study, 50.8–62.3% of the assimilates for grain filling were generated through current photosynthesis (Fig. 6E,F), indicating that dry matter accumulation after anthesis had a greater effect on increasing rice yield^28^. An expanded sink capacity is a key factor underlying the yield advantage of modern high-yield rice cultivars, and this also creates a higher demand for assimilates during the grain-filling stage^29^. Increased N accumulation after anthesis is crucial for grain yield improvement^11^. In the present study, the content of soil available N in the pots of the *Indica-Japonica* hybrid YY12 decreased rapidly after anthesis, while that in the pots of the *Indica* hybrid ZZY1 increased during grain-filling stage (Fig. 1). This clearly demonstrated the difference in soil N demands between the two genotypes after anthesis. At the same time, the N uptake (Post-NU) of YY12 and ZZY1 was 166.6–243.9 mg plant^−1^ and 51.2–105.4 mg plant^−1^, respectively, and the ratio to total N accumulation (Post-NR) was 17.7–26.5% and 6.7–12.8%, respectively (Fig. 5), resulting a 6.0–8.4% difference in grain yield between the two cultivars (Table 1).

**Figure 7:**
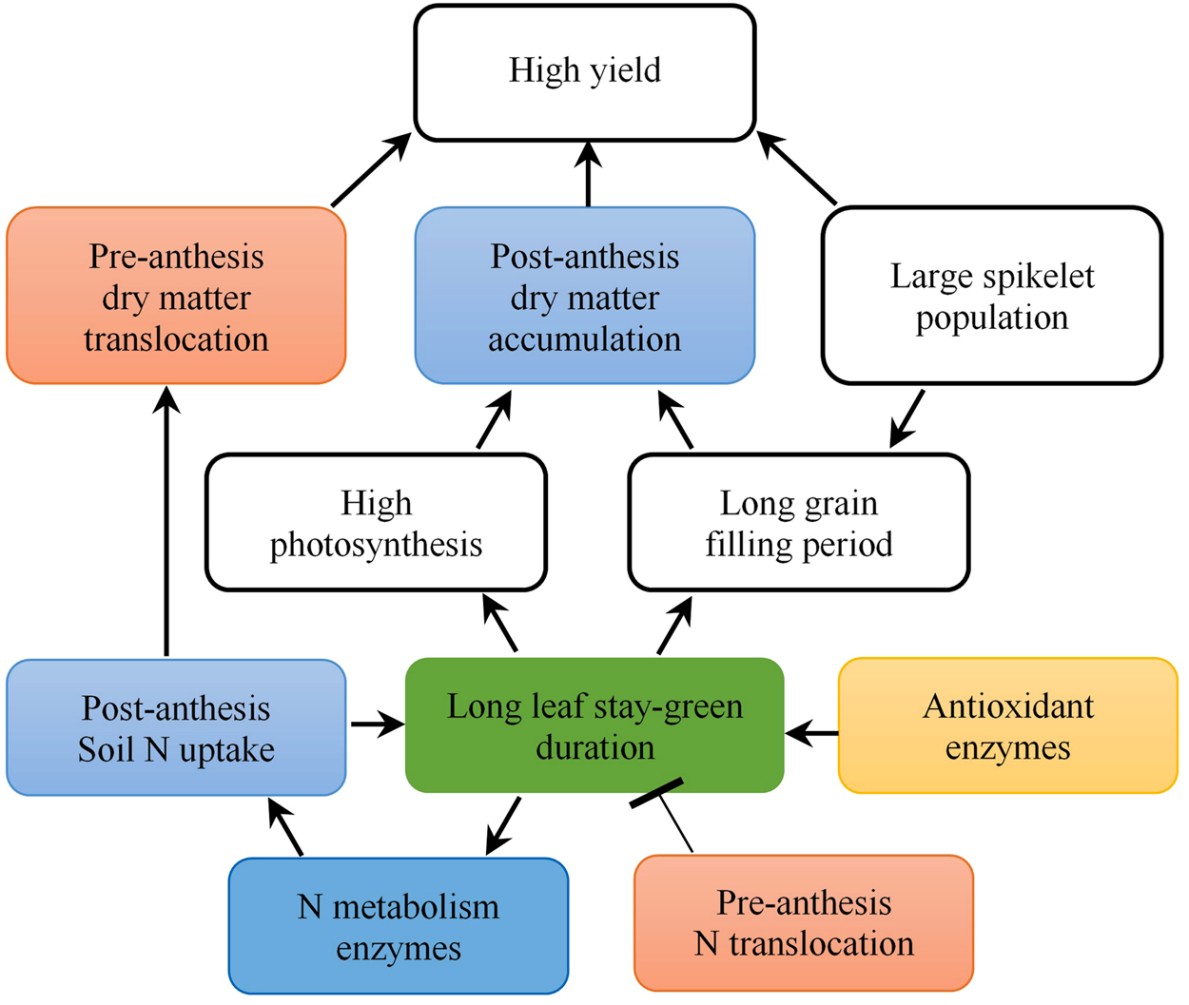
Descriptive model of the roles of post-anthesis nitrogen uptake and translocation in grain yield development. Arrow “→” indicates induction or promotion, while bar “˧” indicates inhibition.

The leaf is not only the main photosynthetic organ but also the main organ for N storage. It was reported that 40–65% of above-ground N is stored in rice leaves at heading^30,31^, while two rice genotypes distributed 43.3–45.5%(ZZY1) and 42.6–48.1% (YY12) in leaves in this study respectively, and total N accumulation and N stored in the leaves of YY12 were significantly higher than those of ZZY1 (Fig. 1G,H). Nitrogen-metabolism enzymes play an important role in the absorption and assimilation of soil N. In higher plants, NR catalyses the reduction of nitrate to nitrite with pyridine nucleotide during N assimilation^32^. The GS/GOGAT cycle is the main pathway of ammonium assimilation in higher plants, and approximately 95% of NH_4_^+^ assimilation occurs via this cycle^33,34^. In this study, when the soil N was sufficient during grain filling, the higher activities of N-metabolism enzymes in leaves promoted N uptake and assimilation from the soil (Fig. 2). However, when the soil N supply was insufficient, the enzyme activities decreased significantly. An increase in GDH activity provides ammonium that can re-enter the N cycle to meet the N demands during grain filling^35^. Higher activities of N-metabolism enzymes increased post-anthesis N uptake, thereby allowing leaves to stay green for longer. This meant that N-metabolism enzymes were retained and remained active for longer, which further promoted N absorption and formed a beneficial cycle (Fig. 7). The strong and active root systems of high-yield rice are another reason for the high N uptake at the grain-filling stage, but the main reason was that the grain-filling period was long enough for sufficient N absorption, rather than relying on the N uptake rate after anthesis^22^.

A longer duration of the green-leaf period means there is more time for photosynthesis, and more temperature and radiation resources are available to satisfy the huge demands of carbon assimilation after anthesis^3^. Modern super-rice cultivars have many spikelets and a long grain-filling period of up to 2 months or more^4,8^. Besides improving the photosynthetic capacity, a long green-leaf period delays leaf senescence and prolongs the period of effective photosynthesis, but it requires a large amount of N uptake after anthesis. It had been reported that higher rates of nutrient accumulation and remobilization resulted in greater leaf area and a delay of leaf senescence in rice^36,37^. This research showed that the higher the N uptake after anthesis, the higher the chlorophyll content and Pn of the functional leaves (Fig. 4) and dry matter accumulation during grain filling (Fig. 6C), although it had little effect on total dry mass per plant at maturity (Table 1). In contrast, the N content and photosynthetic function of the leaves decreased significantly when the N supply was insufficient (Fig. 3). At the same time, the leaf MDA content increased significantly (Fig. 4) and leaf senescence was accelerated, which shortened the duration of the photosynthetic period. Carbon is the main dry matter source for grain filling, and carbon assimilation decreased after anthesis, which affected grain plumpness and grain weight. Antioxidant enzymes also play important roles in delaying senescence and prolonging the stay-green period of leaves^18^. We observed that a high capacity to scavenge reactive species resulted in lower membrane lipid peroxidation and a prolonged stay-green period of leaves (Fig. 4). This was most obvious in YY12.

The N in grains is mainly derived from translocation of N accumulated before anthesis and N uptake from soil after anthesis^31,38^. It was reported that a major proportion of grain N was redistributed from vegetative organs to panicles during grain filling^4,10^. In *Japonica* rice and *Indica* rice grown in the Mediterranean region, the Pre-NTE ranged from 44.7% to 66.7%^19^, compared with 75.4–83.1% (ZZY1) and 56.1–68.8% (YY12) in this study (Fig. 5B). In another study, 64% of the pre-anthesis N translocation was derived from the leaves^20^, while excessive loss of leaf N led to leaf senescence^21^ and shortening of the photosynthetic period. Our results confirmed that a deficiency in N uptake after anthesis accelerated the transport of stored carbon and N, decreased the photosynthetic capacity, accelerated leaf senescence, reduced post-anthesis dry matter assimilation, and decreased the 1000-grain weight, which was the main cause of yield reduction. Nitrogen uptake after anthesis in ZZY1 was only one-third to one-half of that in YY12 (Fig. 5C). Although post-anthesis N deficiency in ZZY1 also reduced the photosynthetic capacity, photosynthetic duration, and 1000-grain weight, it had a much smaller effect on grain yield than in YY12 (Table 1). The meiosis stage is a critical period for rice yield formation. A poor soil N supply at this stage reduced not only the 1000-grain weight of YY12, but also the number of spikelets per panicle, which is highly detrimental to rice genotypes that achieve a high yield through producing a large spikelet population (Table 1).

As discussed above, high activities of N-metabolism enzymes and antioxidant enzymes and low pre-anthesis N translocation were related to high N uptake after anthesis, inhibition of leaf senescence, increased photosynthetic capacity, and a prolonged leaf stay-green period so as to take full advantage of light and temperature resources. These characteristics conferred a strong advantage for post-anthesis dry matter accumulation (Fig. 7). Furthermore, large N uptake after anthesis also promoted the translocation of pre-anthesis stored dry matter instead of N, which could realize the yield potential based on the huge spikelet population. In contrast, weak N uptake after anthesis enhanced the excessive translocation of accumulated N before anthesis, accelerated leaf senescence, reduced the assimilation capacity, and decreased the period of carbon and N assimilation after anthesis, which led to insufficient grain filling and lower yield.

## Materials and Methods

### Plant material and growth conditions

This experiment was conducted from May to November in 2015 and 2016 at the China National Rice Research Institute, Hangzhou (30°04′N, 119°55′E), China. *Indica-Japonica* hybrid rice Yongyou12 (YY12) and *Indica* hybrid rice Zhongzheyou1 (ZZY1) were used as the experimental materials. Pre-germinated seeds were sown in a bowl tray (162 points) covered with a special rice transplanting seedling substrate, with two seeds per point. The 22-day-old seedlings were transplanted into pots (1 plant per pot, each pot 25.5 cm long, 20.0 cm wide, 26.5 cm high, and the volume 13.5 L) with the nitrogen leaching structure as the following description.

The pots were placed in an open space under natural environmental conditions. Alternate wetting and drying irrigation was conducted throughout the cropping season. To each pot, 3.0 g urea was supplied at planting (40%), tillering (30%), and panicle initiation (30%); 3.0 g calcium superphosphate (P_2_O_5_) was applied 1 day before transplanting; and 2.0 g potassium chloride (KCl) was applied at tillering (50%) and panicle initiation (50%). Pests, diseases, and weeds were intensively controlled. Other management practices for high grain yield cultivation were in accordance with local recommendations. All the main stems of the plant were labelled to determine the development stage. The main growth periods of the tested varieties are shown in Table 2.

**Table 2:** Development stages and growth periods of tested varieties in 2015 and 2016.

Year	Variety	Sowing (month/day)	Transplanting (month/day)	Anthesis (month/day)	Maturity (month/day)	Duration from anthesis to maturity (d)	Growth period (d)
2015	Zhongzheyou1	5/22	6/13	8/25	10/5	41	136
	Yongyou12	5/22	6/13	9/3	11/3	61	165
2016	Zhongzheyou1	5/17	6/8	8/20	9/28	39	134
	Yongyou12	5/17	6/8	9/4	11/3	60	170

### Nitrogen leaching structure and treatments

**Figure 8:**
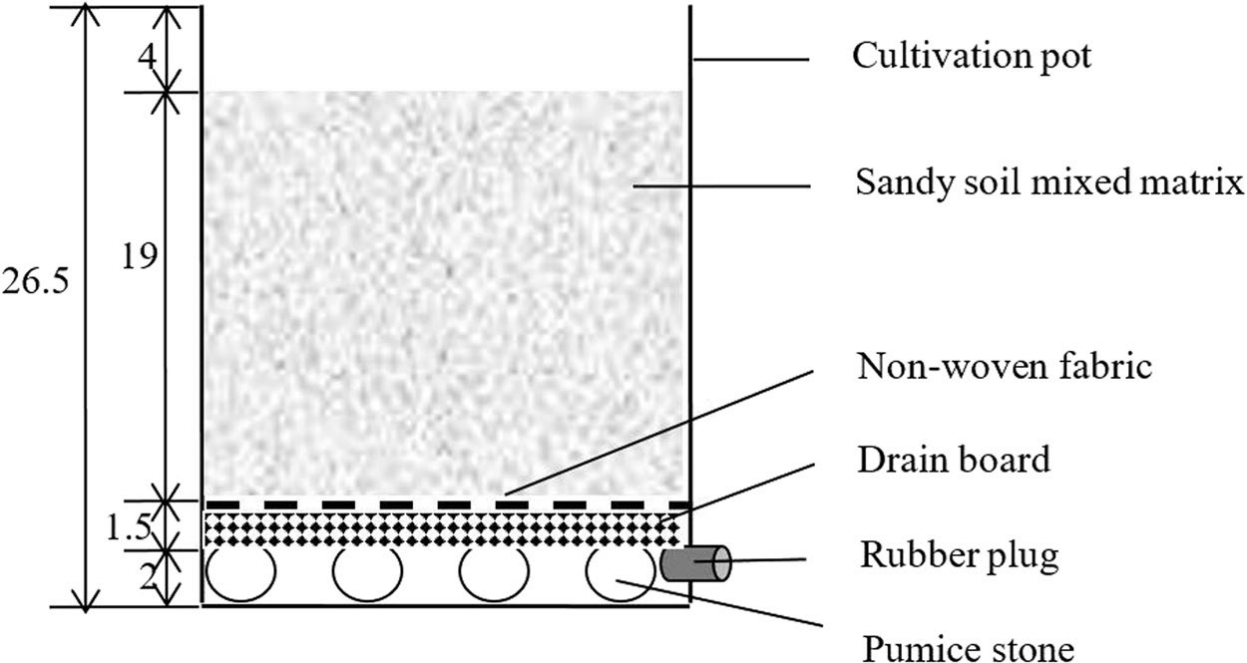
Nitrogen leaching structure in potted soil. Values show length of each part of leaching structure (cm).

The leaching system in the potted soil was created by layering different substrates. The substrates were arranged, from the bottom to the top of each pot, as follows: pumice stone, drainage board (PVC material), non-woven fabric (40 g·m^−2^), 10.0-kg sandy/soil mixed substrate (1:4 ratio) (Fig. 8). The drainage hole at the base of the pot was blocked by a rubber plug. Several pumice stones were placed at the bottom of the pot, and a suitable draining board with many drainage holes was placed on top of the stones. To prevent the soil from plugging the drain hole during the leaching process, the surface of the draining board was covered with two layers of non-woven fabric.

In 2015, plants at the same developmental stage were selected for the AST leaching treatment. Non-treated plants served as the control. In 2016, the plants at the same developmental stage were selected for the leaching treatments at MST and at AST. Non-treated plants served as the control.

One day before the leaching treatment, each pot was placed in a turnover box (25 cm long, 18 cm wide, and 8 cm high), and irrigated with 1 L water to ensure soil moisture saturation. The rubber plug was removed on the next afternoon to start the first leaching. After 12 hours, the rubber plug was replaced, and 1 L water was added to each pot. A second leaching was started 12 hours later. This process was repeated three times in total with 50 pots in each treatment. All the pots were slightly inclined during the leaching process so that the leachate could drain freely. To reduce the evaporation of the leachate under high daytime temperatures and the loss of ammonia by volatilization, all the pots were placed under shelter, and leaching treatments were carried out at night.

### Sampling and measurement of soil nitrogen

Soil samples were collected as cores (0–15 cm depth) from each pot at 14, 11, and 3 d before anthesis, at anthesis (0 d), and at 10, 20, and 37 (maturity stage of ZZY1) or 40 d (YY12), and at 60 d (maturity stage of YY12) after anthesis. The concentration of soil ammonium-N and nitrate-N were determined using the method of Mizota^39^, and the total N content of soil samples was determined with a Kjeltec^TM^ 2400 Kjeldahl meter (FOSS Ltd. Warrington, UK). Five replicates (5 pots) were analysed at each sampling time. At the same time, flag leaves of five main stems were frozen in liquid N for 30 minutes and then stored at −80 °C for further analyses of N-metabolism and antioxidant enzymes.

### Photosynthetic and chlorophyll content measurements

At the anthesis stage (0d), 20 d, 35 d (ZZY1) and 40 d (YY12) after anthesis, the net photosynthetic rate (Pn) of the flag leaf of the labelled main stems was measured in both 2015 and 2016 with a Li-6400 portable photosynthesis system (LI-COR, Lincoln, NE, USA) at PPFD of 1000 *μ*mol m^−2^ s^−1^, leaf temperature of 30 °C, and mass flow of 0.3 mol m^−2^ s^−1^. Five representative plants in each treatment were selected randomly at each time point. The chlorophyll content of the flag leaf was measured with a SPAD (Soil Plant Analysis Development) meter (Konica Minolta, Osaka, Japan)^40^ at the same time as soil sampling, with 10 leaves as 10 replicates.

### Determination of nitrogen-metabolism enzyme activities

Samples of 0.1 g frozen leaf were powdered in liquid N_2_ and homogenized with 1 ml 50 mmol Tris-HCl buffer (pH 8.0) containing 2 mmol Mg^2+^, 2 mmol DTT, and 0.4 mol sucrose. The homogenate was centrifuged at 8,000 g for 10 min at 4 °C, and the supernatant was used for determination of the activities of NR, GS, GOGAT, and GDH. All spectrophotometric analyses were conducted using a multilabel plate reader (Infinite M200-Pro, Tecan, Milan, Italy).

The activity of NR was was assayed as described by Ahmad^32^. The reaction mixture containing 0.25 ml 100 mmol L^−1^ potassiumphosphate buffer (pH 6.8), 0.25 ml 10 mmol L^−1^ KNO_3_, 0.25 ml pyridine nucleotides (NADH, 50 mmol L^−1^); and 0.25 ml enzyme extract was incubated at 33 °C for 30 min. After the completion of incubation period, the reaction was stopped by the addition of zinc acetate and the nitrite produced was estimated chromometrically. One unit of enzyme was described as that amount which catalyzed the reduction of 1 μmol NO_3_^−^ h^−1^ g^−1^ FW.

The activity of GS was determined as described in Zhang^41^ with some modifications. The reaction mixture contained hydroxylamine hydrochloride buffer (pH7.4). After incubation of the mixture at 37 °C for 30 min, the reaction was terminated by adding acidic FeCl_3_ (0.37 mol L^−1^ FeCl_3_ and 0.2 mol L^−1^ TCA in 0.6 mol L^−1^ HCl). Samples were centrifuged at 8000 rpm for 10 min, and the absorbance at 540 nm (A540) was measured chromometrically. The blank was absence of hydroxylamine hydrochloride, and One unit of GS activity was defined as the amount of enzyme catalysing the formation of 1 *µ*mol *γ*–glutamyl hydroxamate per hour at 37 °C.

The activity of NADH–GOGAT was assayed at 30 °C as the method described in Singh and Srivastava^42^. The reaction mixture consisted of 10 mmol a-ketoglutarate, 1 mmol potassium chloride, 37.5 mmol Tris-HCl buffer (pH 7.6), 0.6 mmol nicotinamide adenine dinucleotide (NADH), 8 mmol L-glutamine and 0.3 ml enzyme. The absorbance at 340 nm was monitored for 300 s. The activity of NADH–GOGAT was estimated using the molar extinction coefficient of NADH, and one unit was defined as the amount reducing 1 nmol NADH per minute at 30 °C.

The activity of GDH activity was determined using the method of Masclaux^43^. The reaction mixture contained 300 mmol Tris-HCl buffer (pH 8.0), 600 mmol ammonium chloride, 3 mmol calcium chloride, 0.6 mmol NADH, and 0.1 mL enzyme. The reaction was started by adding enzyme extract and carried out at 30 °C. The absorbance at 340 nm was monitored for 300 s, and the activity of GDH was expressed as nmol NADH·per minute at 30 °C.

### Determination of malondialdehyde content and antioxidant enzyme activities

To determine SOD and CAT activities, about 0.1 g frozen leaf tissue was ground at 4 °C in a mortar with 5 ml 50 mmol phosphate buffer solution (pH 7.8) containing 1% PVP. The homogenate was centrifuged at 8,000 g for 20 min.

The SOD activity was estimated by measuring its ability to inhibit the photochemical reduction of nitroblue tetrazolium (NBT) by 50%^44^. The SOD reaction system contained 25 mmol sodium phosphate buffer (pH7.8), 13 mmol methionine, 2 mmol riboflavin, 10 mmol EDTA-Na_2_, 75 mmol nitro blue tetrazolium (NBT), and modest amount extract. Samples were put under light (300 mmol m^−2^ s^−1^) for 20 min. The photo-reduction of NBT was measured at 560 nm, and SOD activity was expressed as U g^−1^ FW.

CAT activity was determined after the reaction of the extract in the presence of 50 mmol sodium phosphate buffer (pH7.0) and 20 mmol H_2_O_2_ (3 mL). The reaction was carried out at 30 °C, and the absorbance at 240 nm was monitored for 300 s^45^. CAT activity was calculated according to the molar extinct coefficient of H_2_O_2_ and expressed as *μ*mol H_2_O_2_ g^−1^ FW min^−1^.

The MDA content and APX activity were determined using a commercial chemical assay kit (Jiangsu Keming Biotechnology Institute, Suzhou, China). For analyses of MDA content and APX activity, about 0.1 g frozen leaf tissue was homogenized in 1 ml buffer I [50 mmol phosphate buffer (pH 7.8), containing 0.1 mmol EDTA, 0.5% (*w/v*) Triton-100 and 2% PVP], which was supplied in the assay kit, at 4 °C with a mortar and pestle. The mixture was centrifuged at 8,000 g at 4 °C for 10 min and the supernatant was used for MDA and APX analyses according to the manufacturer’s instructions. All spectrophotometric analyses were conducted using a multilabel plate reader (Infinite M200-Pro, Tecan, Milan, Italy).

### Dry matter and total nitrogen of plants

At the anthesis stage (0 d), and 10, 20, and 37 (maturity stage of ZZY1) or 40 d (YY12), and at 60 d (maturity stage of YY12) after anthesis, sampled plants were divided into the leaf lamina, sheath plus stem, and panicle. The plant parts were dried to constant weight and ground into a powder. Approximately 0.20 g powder was digested with H_2_SO_4_ at 260 °C for measurement of total nitrogen with a KjeltecTM 2400 Kjeldahl meter (FOSS Ltd. Warrington, UK). Plants were harvested from five pots at each sampling time (five replicates). The dry matter (DM) values were used to calculate and analyse dry matter production and distribution, and the total nitrogen values were used to calculate and analyse total nitrogen uptake and translocation.

### Yield and its components

The filled and unfilled spikelets were separated using a seeds air separator CFY-II (Zhejiang Top Instrument Co., Ltd., China), and then counted and weighed. These values were used to calculate the number of spikelets per panicle, seed-setting rate (%), and 1000-grain weight (g). Grain yield per plant (g), at an adjusted moisture content of 14% (YY12) or 13.5% (ZZY1) FW, was determined from five pots.

### Data analysis

Data were calculated on the basis of DM and measured N values, and parameters were calculated as follows^19,38^:

- Pre-anthesis DM translocation (Pre-DMT, g plant^−1^) = Total aboveground DM at anthesis − DM of vegetative parts (leaves and stems) at maturity.
- Pre-anthesis DM translocation efficiency (Pre-DMTE, %) = Pre-DMT/Total aboveground DM at anthesis × 100.
- Post-anthesis DM accumulation (Post-DMA, g plant^−1^) = Total aboveground DM at maturity − Total aboveground DM at anthesis.
- Ratio of post-anthesis DM accumulation to total DM accumulation (Post-DMR, %) = Post-DMA/Total DM accumulation × 100.
- Contribution of pre-anthesis translocation or post-anthesis accumulation of DM to grain yield (DMC, %) = Pre-DMT or Post-DMA/Grain weight at maturity × 100.
- Pre-anthesis N translocation (Pre-NT, mg plant^−1^) = Total aboveground N at anthesis − N of vegetative parts at maturity.
- Pre-anthesis N translocation efficiency (Pre-NTE, %) = Pre-NT/Total aboveground N at anthesis × 100.
- Post-anthesis N uptake (Post-NU, mg plant^−1^) = Total aboveground N at maturity − Total aboveground N at anthesis.
- Ratio of post-anthesis N uptake to total N accumulation (Post-NR, %) = Post-NU/Total N accumulation × 100.
- Contribution of pre-anthesis translocation or post-anthesis uptake of N to grain yield (NC, %) = Pre-NT or Post-NU/Grain N at maturity × 100.

Data are presented as means ± SE. Differences among the treatments were determined based on Tukey’s multiple test (*p* < 0.05) with SPSS 23.0 software.

## Data Availability

The data used or analysed during the current study are available from the corresponding author on reasonable request.
